# No Tömösváry organ in flat backed millipedes (Diplopoda, Polydesmida)

**DOI:** 10.3897/zookeys.930.48438

**Published:** 2020-04-28

**Authors:** Leif Moritz, Markus Koch

**Affiliations:** 1 Zoologisches Forschungsmuseum Alexander Koenig, Leibniz Institute for Animal Biodiversity, Section Myriapoda, Adenauerallee 160, 53113, Bonn, Germany Zoologisches Forschungsmuseum Alexander Koenig Bonn Germany; 2 Institute of Evolutionary Biology and Ecology, University of Bonn, An der Immenburg 1, 53121, Bonn, Germany University of Bonn Bonn Germany; 3 Senckenberg Gesellschaft für Naturforschung, Dept. Information Technology and Biodiversity Informatics, Senckenberganlage 25, 60325, Frankfurt am Main, Germany Senckenberg Gesellschaft für Naturforschung Frankfurt am Main Germany

**Keywords:** Anatomy, Histology, Micro-Computed Tomography, Morphology, Tentorium

## Abstract

The Tömösváry organ is a sensory structure of the head in myriapods and some other terrestrial arthropods. Due to its variable shape, size, and position in millipedes (Diplopoda) the Tömösváry organ is commonly used as diagnostic character in taxonomic descriptions and often included in phylogenetic analyses. For the Polydesmida, the largest millipede order, the Tömösváry organ is inconsistently stated as being either absent or present as a pear-shaped pit covered by a membrane or cuticular disc. In order to resolve this inconsistency, we investigated the morphology of the presumable Tömösváry organ in four polydesmidan species based on paraffin-histology, semi-thin sections and micro-computed tomography. Our results unambiguously favor the view that the articulation of the cephalic tentorium with the head capsule was misidentified as the Tömösváry organ in previous studies, and thus that the Tömösváry organ indeed is absent in the Polydesmida. The pear-shaped pit proved to represent the distal roundish expansion of the incisura lateralis, to which – similarly as in julidan millipedes – the tentorial transverse bar is articulated. The absence of the Tömösváry organ in the Polydesmida does not affect the topology of the interrelationships among the millipede orders retrieved in previous cladistic analyses based on morphology. As a character shared by Colobognatha and Juliformia, however, absence of a Tömösváry organ in Polydesmida favors the optimization of its presence in nematophoran millipedes as a reversal. Further studies are needed to clarify whether among chilognathan millipedes a Tömösváry organ really exists in taxa such as Stemmiulida, and whether the Tömösváry organs are homologous across millipedes.

## Introduction

The Tömösváry organ is a paired sensory organ, situated on the head of millipedes (Diplopoda) and other Myriapoda posterior of the antennal base. It is also referred to as postantennal organ (e.g., Altner and Thies 1976), temporal organ (e.g., Bedini and Mirolli 1967; Yamana et al. 1986) or in German as either “Schläfenorgan” (e.g., Tömösváry 1883; Latzel 1884; Verhoeff 1926–1928; Seifert 1932) or “Schläfengrube” (e.g., Vom Rath 1886) or as “foveae lateralis capitis” (e.g., Vom Rath 1886). The function of the Tömösváry organ is still unclear and several competing hypotheses exist, as discussed in detail by Müller and Sombke (2015), such as reception of vibration (e.g., Pflugfelder 1933; Meske 1961), olfaction (e.g., Zograf 1899; Bedini and Mirolli 1967), gravitation (e.g., Krishnan 1968), or humidity (e.g., Bedini and Mirolli 1967).

Tömösváry organs can be present in various shapes, forming a groove, pit or tube (Hennings 1906; Müller and Sombke 2015), and possess a sensory cavity with a thin and porous cuticle formed by a peg-like or hemispherical epidermis (Müller and Sombke 2015). In addition to studies on the physiology and morphology of the Tömösváry organ it is also used as an important taxonomic and phylogenetic character due to the variation in its shape, position or size (e.g., Hennings 1906; Attems 1926; Sierwald and Bond 2007; Blanke and Wesener 2014; Müller and Sombke 2015; Bouzan et al. 2017a, b). This is also true for the Polydesmida, where it has been coded in phylogenetic analyses as present and small (Blanke and Wesener 2014, characters 6 and 7) or as small pit (Sierwald and Bond 2007, character 18).

The order Polydesmida is the most diverse order of the millipedes (Diplopoda), with more than 5000 described species (Brewer et al. 2012) in 30 families (Shelley 2002), which contribute to over a third of the more than 12,000 known millipede species. The flat body of the blind Polydesmida consists of usually 17 or 18 fully fused body-rings (plus one apodous ring and telson) with large paranota (Enghoff et al. 2015), a habitus known as litter-splitter (Golovatch and Kime 2009).

There is uncertainty about the absence or presence of the Tömösváry organ in the Polydesmida. Its presence in Polydesmida was first stated by Attems (1899) and subsequently reported by him repeatedly (e.g., Attems 1926, 1937). Since the first detailed description of the presumable Tömösváry organ in the Polydesmida by Hennings (1906), this peculiar organ has not been re-examined for the order. Attems (1926) questioned its presence in some genera of the Polydesmida (Attems 1926, p. 55), while Verhoeff (1926–1928) depicted the Tömösváry organ for *Coromus thomsonii* (Verhoeff 1926–1928, p. 771, fig. 364), and Snodgrass (1952) described a Tömösváry organ for *Apheloria coriacea*. Seifert (1932), in contrast, stated its absence in the Polydesmida (Seifert 1932, p. 436), without referring to previous records. Apparently Richard L. Hoffman likewise affirmed absence of the Tömösváry organ in the Polydesmida in an unpublished manuscript (P. E. Marek, pers. comm. December 2019). Although it seems to be mostly accepted among some taxonomic experts that the Tömösváry organ is absent in the Polydesmida (W. A. Shear and P. E. Marek, pers. comm. 12^th^ May 2017) it is still stated as being present in recent cladistic analyses and taxonomic descriptions (e.g., Sierwald and Bond 2007; Blanke and Wesener 2014; Enghoff et al. 2015; Müller and Sombke 2015; Bouzan et al. 2017a, b), usually with reference to Hennings (1906) or Attems (1926).

In this study we aim to clarify whether the Tömösváry organ is present or absent in the Polydesmida, and which structure was originally described by Hennings (1906).

## Material and methods

### Specimens and data deposition

Four species representing four families (Polydesmidae, Paradoxosomatidae, Oxydesmidae, Gomphodesmidae) and three suborders (Polydesmidea, Strongylosomatidea, Leptodesmidea) were investigated. Specimens of *Polydesmus angustus* (Latzel, 1884) were collected in April 2015 in the Kottenforst (50°41'05.3"N, 07°05'19.4"E, Bonn, Germany) and fixed in Bouin-solution for paraffin-histology and micro-CT scanning, or in Karnovsky fixative (2.5% glutaraldehyde, 3.2% paraformaldehyde in 0.1M salted phosphate buffer, pH 7.2) for semi-thin sections. Furthermore, for investigations with micro-CT only, specimens of *Oxidus gracilis* (Koch, C. L., 1847) (collected in the Botanical Garden of the University of Bonn, Germany in April 2018), *Coromus vittatus* (Cook, 1896) (obtained via the pet trade from Nigeria) and *Tymbodesmus* sp. (obtained via the pet trade from Cameroon) were fixed in Bouin-solution. Micro-CT data and histological images of *Polydesmus angustus* are deposited on Morphobank (O’Leary and Kaufman 2011, 2012) under project number 3582 (http://morphobank.org/permalink/?P3582). The specimens studied by micro-CT are stored in the Zoological Research Museum Alexander Koenig (ZFMK) as vouchers (see Table 1).

**Table 1.** Taxon sampling and scanning parameters for micro-computed tomography.

	ID	Location	Voltage	Current	Pixel size	Exposure	Rotation steps	Rotation	Averaging
***Polydesmus angustus***	ZFMK-MYR08922	Kottenforst, Bonn, Germany	40 kV	200 µA	2.6 µm	1659 ms	0.1°	180°	7
***Oxidus gracilis***	ZFMK-MYR08923	Botanical garden, Bonn, Germany	50 kV	200 µA	1.2 µm	500 ms	0.1°	180°	7
***Coromus vittatus***	ZFMK-MYR08924	Pet trade, Nigeria	60 kV	166 µA	3.6 µm	500 ms	0.1°	180°	7
***Tymbodesmus* sp.**	ZFMK-MYR08925	Pet trade, Cameroon	43 kV	200 µA	1.8 µm	1800 ms	0.15°	360°	10

### Histology

Following Hennings (1906) histological sections were acquired of specimens embedded in paraffin wax. Specimens fixed with Bouin-solution were decalcified in 5% nitric acid for 6 hours before embedding in paraffin (Paraplast, Sigma-Aldrich). Sections with a thickness of 7 µm were obtained with a Leica RM2165 microtome and stained with a trichrome Azan-staining. To obtain semi-thin sections, specimens fixed in Karnovsky fixative for one hour and decalcified in 5% nitric acid were embedded into an Epon-Araldite epoxy resin (Electron Microscopy Science). Semi-thin sections of the specimen’s head with a thickness of 1 µm were made with a Diatome Histojumbo Hj4237 diamond knife at a Reichert Ultracut S ultramicrotome (Leica). The semi-thin sections were stained with Toluidine blue. Sections were photographed with a dotSlide Olympus BX51 light microscope and the software dotSlide 2.5 (Olympus Soft Imaging Solutions GmbH). The digital images were aligned in an image stack with the software Imodalign (B. Quast, https://www.q-terra.de/biowelt/3drekon/tools/imodalign/imodalign.htm).

### Micro-computed tomography (micro-CT) and visualization

For micro-CT scanning one specimen each fixed in Bouin-solution of *Polydesmus angustus* (Latzel, 1884), *Oxidus gracilis* (Koch, C. L., 1847), *Coromus vittatus* (Cook, 1896) and *Tymbodesmus* sp. were transferred to 96% ethanol via an ascending ethanol series and stained with 3% Iodine solution for 24 hours. The specimens were washed in 100% ethanol and critical point dried using a Leica EM CPD 300. Micro-CT scanning was performed at the ZFMK using a SKYSCAN 1272 (Bruker micro-CT) with random movement = 15 and flat-filed correction and geometric correction switched on. For varying scanning parameters see Table 1. Post-alignment, ring-artefact reduction, beam-hardening correction and reconstruction were performed in NRecon 1.7.1.6 (Bruker microCT). The image stacks were modified using Fiji ImageJ 1.50e (Schindelin et al. 2012). Volume rendering was performed in Drishti Version 2.6.3 (Limaye 2012). Segmentation was done in ITK-SNAP 3.6.0 (Yushkevich et al. 2006). Images were edited in GIMP version 2.10.6 (https://www.gimp.org) and Inkscape 0.92 (www.inkscape.org).

## Results

**Figure 1.**
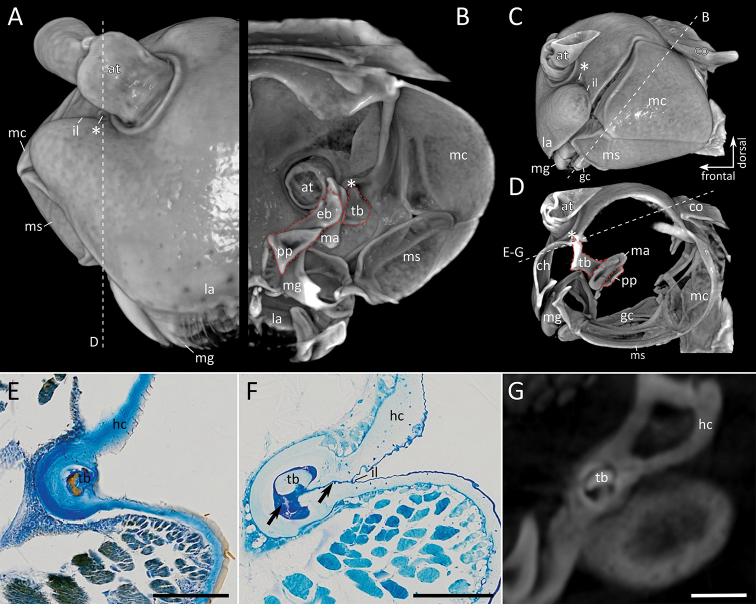
*Polydesmus angustus*, head **A–D** volume rendering based on micro-CT data: **A** Frontal view **B** cross-section, posterior view, plane indicated in C **C** lateral view **D** sagittal view, cutting plane indicated in A **E–G** details of connection of tentorial transverse bar to head capsule at incisura lateralis, plane as indicated in D: **E** histological section (Paraffin, Azan-staining) **F** histological section (Araldite, Toluidine blue) **G** optical section of micro-CT scan. Abbreviations: at = antenna, co = collum, eb = epipharyngeal bar of tentorium, gc = gnathochilarium, gls = gnathal lobe sclerite, hc = head capsule, il = incisura lateralis, la = labrum, mc = mandibular cardo, mg = mandibular gnathal lobe, ms = mandibular stipes, pp = posterior process of tentorium, tb = transverse bar of tentorium. Asterisk (*) indicates structure previously interpreted by Hennings (1906) as the Tömösváry organ in the Polydesmida. In the volume renderings the tentorium is marked with a red dotted line. Scale bar: 100 µm (**E–G**).

**Figure 2.**
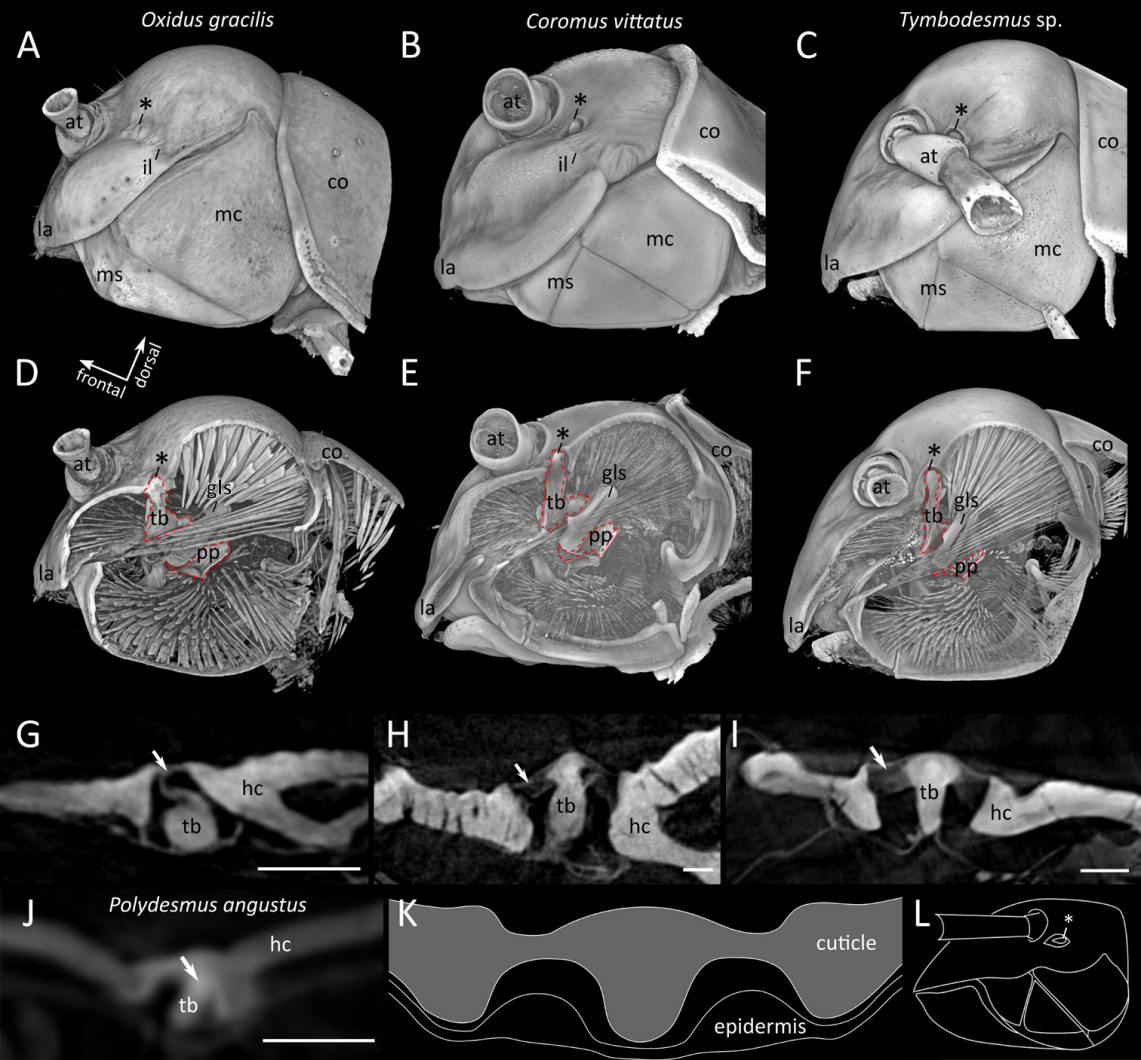
Articulation of the tentorial transverse bar to the head capsule in the Polydesmida**A–C** head in lateral view (anterior is left), volume rendering **D–F** head, sagittal section through tentorial transverse bar, volume rendering **A, D, G***Oxidus gracilis***B, E, H***Coromus vittatus***C, F, I***Tymbodesmus* sp. **J***Polydesmus angustus***G–J** optical cross sections **K, L** the Tömösváry organ in *Eurydesmus laxus* after Hennings (1906): **K** cross-section, modified from Hennings (1906, plate XXXI, fig. 11) **L** head in lateral view, modified from Hennings (1906, plate XXXI, fig. 9). Abbreviations: at = antenna, co = collum, gls = gnathal lobe sclerite, hc = head capsule, la = labrum, mc = mandibular cardo, ms = mandibular stipes, tb = transverse bar of tentorium, pp = posterior process of tentorium. Asterisk (*) indicates the structure previously interpreted by Hennings (1906) as Tömösváry organ. In the volume renderings the tentorium is marked with a red dotted line. Arrows indicate the flexible connection of transverse bar and head capsule. Scale bars: 100 µm (**G–J**).

In all studied species the structure described as Tömösváry organ (Fig. 1A, *) in previous studies demarcates the distal roundish expansion of the incisura lateralis (Fig. 1B, C). At this point of the incisura lateralis the transverse bar of the tentorium projects through the head capsule. From the outside the tip of the transverse bar appears oval in shape and is surrounded by a rim (Figs 1C; 2A–C) formed by the cephalic cuticle (Figs 1D; 2D–F). The cuticle of the head capsule is soft in this region and surrounds the tip of the transverse bar completely (Figs 1E, F; 2G–J). While in *Polydesmus angustus* and *Oxidus gracilis* the transverse bar does not project over the level of the surrounding cuticle (Figs 1A, D; 2A, D), it is more exposed in *Coromus vittatus* and *Tymbodesmus* sp. (Fig. 2B, C, E, F). No structure similar to a Tömösváry organ in other millipedes is associated with the flexible connection of the transverse bar to the head capsule.

**Figure 3.**
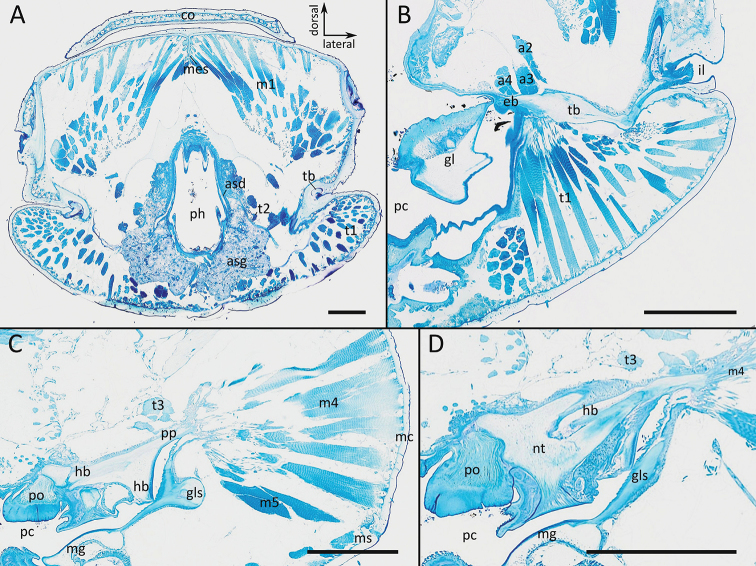
*Polydesmus angustus*, histological sections from anterior (**A**) to posterior (**D**) **A** articulation of tentorial transverse bar to head capsule **B** tentorial transverse bar and epipharyngeal bar **C** tentorial hypopharyngeal bar and posterior process **D** articulation of tentorium to “Nebententorium”. Abbreviations: a2 = medial antennal muscle, a3 = lateral antennal muscle, a4 = anterior antennal muscle, asd = duct of anterior salivary gland, asg = anterior salivary gland, co = collum, eb = epipharyngeal bar of tentorium, gl = mandibular gnathal lobe, gls = gnathal lobe sclerite, hb = hypopharyngeal bar of tentorium, m1 = gnathal lobe sclerite muscle, m4 = anterior mandibular cardo muscle, m5 = posterior mandibular cardo muscle, mc = mandibular cardo, mes = median septum, mg = mandibular gnathal lobe, ms = mandibular stipes, nt = ‘Nebententorium’, pc = preoral chamber, ph = pharynx, po = ‘Presshöcker’, t1 = anterior tentorial muscle, t2 = dorsal tentorial muscle, tb = transverse bar of tentorium. Scale bars: 200 µm.

The general structure of the tentorium of the studied species is the same as described by Seifert (1932) for *Strongylosoma pallipes*. The transverse bar (tb) extends from the incisura lateralis (Fig. 3A) posteriorly and bends mesially off about 90°, where it becomes plate-like. Along its mesal extension towards the preoral chamber the tb serves as insertion for the anterior tentorial muscle (t1) which originates from the head capsule. Antero-laterally the transverse bar passes over into the epipharyngeal bar (eb) (Fig. 3B). On its distal tip the dorsal tentorial muscle (t2) inserts, which originates from the head capsule mesal of t1. Posteriorly the epipharyngeal bar passes over into the hypopharyngeal bar (hb), which is located within the hypopharyngeal wall and distally articulated to the ‘Nebententorium’ (Fig. 3C, D). At the point where epipharyngeal bar and hypopharyngeal bar meet, the plate-like posterior process (pp) projects posteriorly into the head capsule. The posterior process serves as origin of three (medial, lateral and anterior) antennal muscles (a2, a3, a4) inserting on the antennal base, and of the tentorial pharyngeal dilator muscle (p5), which inserts laterally on the pharyngeal wall. Furthermore, the mandibular muscles m4/m5 originate from the posterior margin of the tentorial posterior process and insert at the mandibular base.

Attached on the distal margin of the posterior process is the posterior tentorial muscle (t3), which originates from the postoccipital flange, and the ventral tentorial muscle (t4), which originates from the transverse mandibular tendon (see Suppl. material 1: file S1).

## Discussion

### No Tömösváry organ in Polydesmida

Hennings (1906) described the alleged Tömösváry organ of the polydesmid *Eurydesmus laxus* Gerstaecker, 1873 as a pear-shaped pit covered by a membrane with a median hard swelling (Fig. 2K, L; compare also to Hennings 1906, p. 593). Snodgrass (1952) also described the Tömösváry organ of the Polydesmida as oval groove with a thickened central disc, beneath which sensory cells lie. Such a structure cannot be observed in the studied species. A structure resembling the general appearance of the Tömösváry organ in millipedes, with a sensory cavity lined by a thin porous cuticle and a peg-like epithelium is not present in the Polydesmida. All previous descriptions as well as the accompanying graphical depictions provided by Attems (1899, 1937), Verhoeff (1926–1928) and Snodgrass (1952) for the Polydesmida unambiguously refer to a structure we identified as the projection of the tentorial transverse bar through the head capsule. Snodgrass (1951) stated that the tentorial transverse bar (fulturae sensu Snodgrass 1951) attaches to the central disc of a horseshoe-shaped Tömösváry organ in the Polydesmida, but in the studied species no separate disc-like structure was found. Instead a knob-like distal tip of the tentorial transverse bar is present. Based on our data it is now obvious that the connection of the tentorial transverse bar to the head capsule was misidentified as the Tömösváry organ in Polydesmida. Hennings (1906) stated that the alleged Tömösváry organ only varies in size in the seven species of Polydesmida he studied (*Oranmorpha guerini*, *Orthomorpha coarctata*, *Orthomorpha tenuipes*, *Polydesmus complanatus*, *Spanobrachium collaris*, *Fontaria* sp., *Aphelidesmus uncinatus*), among which is one congener of *Polydesmus angustus* (*P. complanatus*), while being absent in species capable of volvation (i.e., *Lignydesmus rubriceps*, *Oniscodesmus aurantiacus* and *Aporodesmus gabonicus*).We accordingly hypothesize that the Tömösváry organ is generally absent in the Polydesmida.

This conclusion (absence of the Tömösváry organ in the Polydesmida) is further supported by previous doubts on the presence of a nervus tömösváryi in the Polydesmida (Sombke and Rosenberg 2015). The nerve innervating the Tömösváry organ in other myriapods could not be identified either in our histological studies, in contrast to Hennings (1906) who stated its presence, but absence of the nervus opticus. The absence of the alleged Tömösváry organ in *Lignydesmus rubriceps*, *Oniscodesmus aurantiacus* (Hennings 1906) and in *Cyclodesmus* (Attems 1899) can straightforwardly be related to their ability to volvate (Golovatch 2003). In the same context of volvation, the lateral connection of the tentorium to the head capsule via the transverse bar is also lost in Sphaerotheriida (Moritz and Wesener 2017; Moritz et al. 2018).

### Phylogenetic significance of the Tömösváry organ

The absence of the Tömösváry organ in the Polydesmida is a character shared with the Colobognatha, Stemmiulida, Juliformia and Siphoniulida among the chilognathan millipedes (Sierwald and Bond 2007; Blanke and Wesener 2014). The phylogenetic analyses based on morphological data by Sierwald et al. (2003) and Blanke and Wesener (2014) suggest a sister-group relationship of Polydesmida and Nematophora. Other phylogenetic analyses resolve the Polydesmida as more closely related to either Juliformia (Enghoff et al. 1993; Cong et al. 2009), Stemmiulida (Rodriguez et al. 2018), or Colobognatha (Sierwald and Bond 2007), all of which do not possess a Tömösváry organ. Although Silvestri (1903) depicts the Tömösváry organ for the stemmiulid *Stemmiulus ortonedae*, its apparent presence likewise requires re-consideration according to Müller and Sombke (2015). The correction of the character coding related to the Tömösváry organ for Polydesmida in the character matrix compiled by Blanke and Wesener (2014) does not alter its topology, in which the Polydesmida is the sister group of the Nematophora. The resolution of the chilognathan orders in this analysis, however, now questions the homology of the Tömösváry organ across millipedes, since its presence in (some) Nematophora optimizes most parsimoniously as a reversal. Based on the available data, the question of whether the Tömösváry organ among chilognathans is exclusively maintained or instead regained in Nematophora remains an issue of debate. Therefore, detailed investigations of the head morphology for all millipede orders are needed combining various techniques including developmental studies.

## Conclusion

Contrary to several old and recent publications (e.g., Hennings 1906; Verhoeff 1926–1928; Snodgrass 1951, 1952; Blanke and Wesener 2014; Müller and Sombke 2015; Bouzan et al. 2017a, b) the Polydesmida do not seem to possess a Tömösváry organ. Indeed, the connection of the tentorial transverse bar laterally to the head capsule has been misinterpreted as the Tömösváry organ, as we show here. The absence of the Tömösváry organ in the Polydesmida, Juliformia and Colobognatha may be due to multiple losses, but parsimony favors its sole presence in Nematophora among Chilognatha as a reversal. To further clarify the distribution, homology and evolution of the Tömösváry organ in the Diplopoda more detailed studies are needed.

## Supplementary materials

Supplementary material 1 *Polydesmus angustus*, head capsule and tentorium with associated musculature Data type multimedia https://binary.pensoft.net/file/404845This dataset is made available under the Open Database License (http://opendatacommons.org/licenses/odbl/1.0/). The Open Database License (ODbL) is a license agreement intended to allow users to freely share, modify, and use this Dataset while maintaining this same freedom for others, provided that the original source and author(s) are credited.Leif Moritz, Markus Koch
