# Supplementary figures and images for: No Tömösváry organ in flat backed millipedes (Diplopoda, Polydesmida)
Source: Zookeys. 2020 Apr 28;930:103–15. doi: 10.3897/zookeys.930.48438 (PMC7200888; doi:10.3897/zookeys.930.48438)

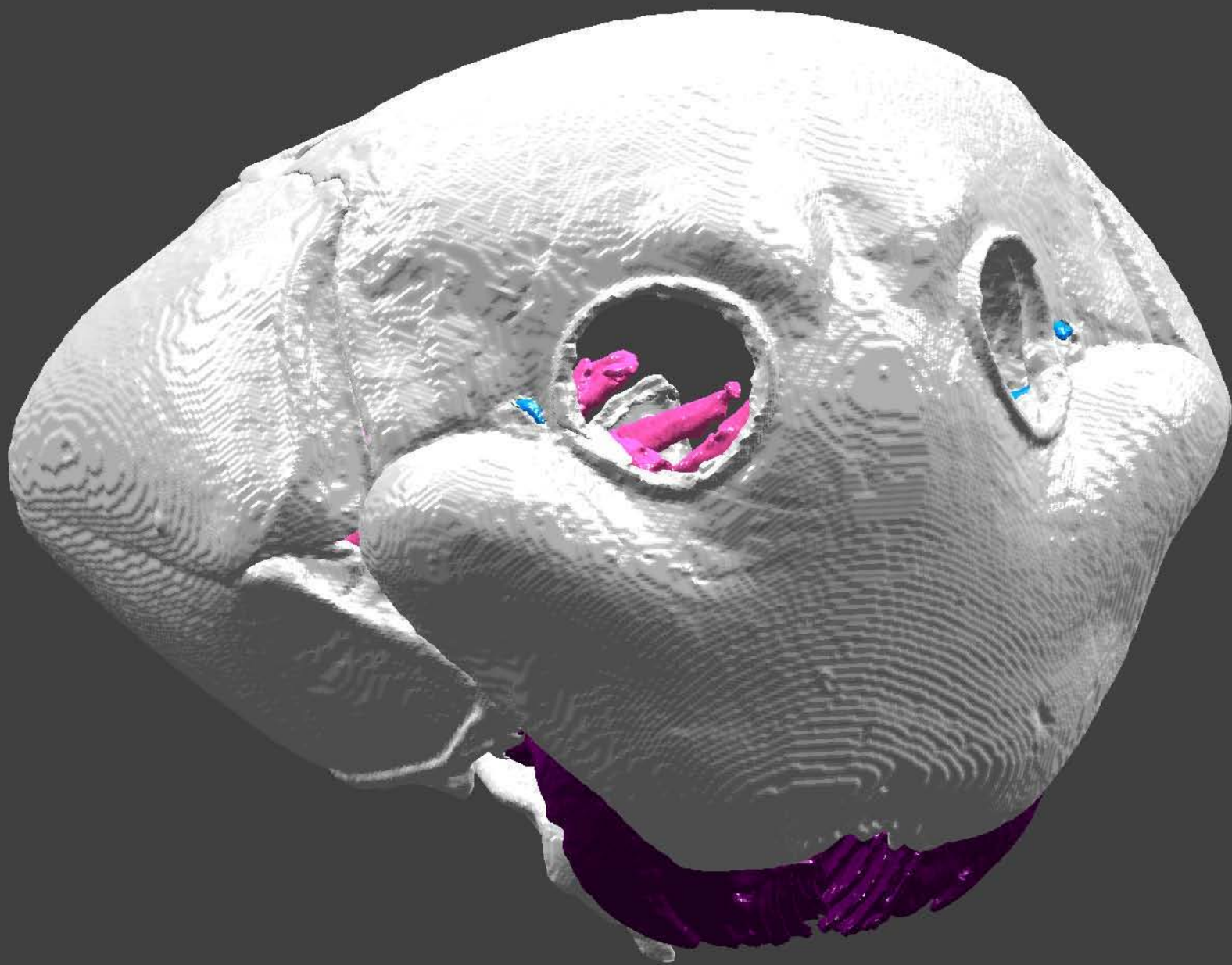

Supplement: Supplementary material 1 — Polydesmus angustus, head capsule and tentorium with associated musculature [file zookeys-930-103-s001.pdf]
